# Pelvic Osteochondroma Causing a Painful Anterior Snapping Hip With Concomitant Femoroacetabular Impingement: A Case Report

**DOI:** 10.7759/cureus.103498

**Published:** 2026-02-12

**Authors:** Molly C Meadows, Gregory Charville, Stephanie Y Pun

**Affiliations:** 1 Orthopedic Surgery, Stanford University, Palo Alto, USA; 2 Surgical Pathology, Stanford University, Palo Alto, USA

**Keywords:** femoroacetabular impingement, hip arthroscopy, internal snapping hip, labral repair, osteochondroma

## Abstract

The differential diagnosis for hip pain in a young patient can present a diagnostic challenge. We present an unusual case of concomitant femoroacetabular impingement (FAI) and painful internal snapping hip caused by an osteochondroma of the superior pubic ramus. The patient was treated with arthroscopic labral repair and femoral osteochondroplasty, and open osteochondroma excision, resulting in complete resolution of both FAI and snapping hip. Pelvic osteochondroma is an uncommon cause of hip pain that can mimic FAI symptoms. Early advanced imaging and combined approaches may be necessary to ensure that all causes of hip pathology are addressed.

## Introduction

The differential diagnosis of anterior hip pain includes both intra- and extra-articular pathology. A common intra-articular diagnosis is femoroacetabular impingement (FAI), which involves impingement between the femoral neck and the anterior rim of the acetabulum [1]. FAI is more common in hips with a cam deformity of the proximal femur, which describes a loss of sphericity of the femoral head with a bony prominence at the femoral head/neck junction leading to impingement against the acetabulum. FAI also occurs in hips with acetabular retroversion and acetabular over-coverage.

A common extra-articular cause of anterior hip pain is a snapping iliopsoas tendon. Painful anterior snapping and locking symptoms occur when the hip is taken from flexion to extension, causing the iliopsoas tendon to roll over the iliopectineal eminence, iliopsoas bursa, or prominent anterior femoral head [2]. Occasionally, patients with FAI also experience a painful snapping iliopsoas tendon, but extra-articular causes of a painful snapping iliopsoas tendon must be ruled out to ensure resolution of all symptoms with appropriate treatment. Thus, a careful workup, including a detailed physical exam, diagnostic injections, and advanced imaging, is often necessary to make the appropriate diagnosis [3].

Here, we describe an unusual mixed presentation of anterior hip pain due to an osteochondroma of the superior pubic ramus causing painful internal snapping hip, and a cam deformity causing symptomatic FAI. The patient was informed that data concerning the case would be submitted for publication, and he provided consent.

## Case presentation

A 19-year-old man presented with complaints of two years of worsening right anterior hip pain associated with locking and catching. He previously played high school football but was unable to play in college due to hip pain. He attempted physical therapy without improvement in his symptoms. His physical examination was notable for pain with resisted hip flexion and a positive anterior impingement test that reproduced his pain. Direct palpation of the iliopsoas muscle also reproduced his pain, and range of motion of the hip re-created the painful snapping of the iliopsoas tendon. His hip range of motion was symmetric to the contralateral hip, with flexion to 90°, internal rotation in flexion limited to 10°, and external rotation in flexion to 60°.

**Figure 1:**
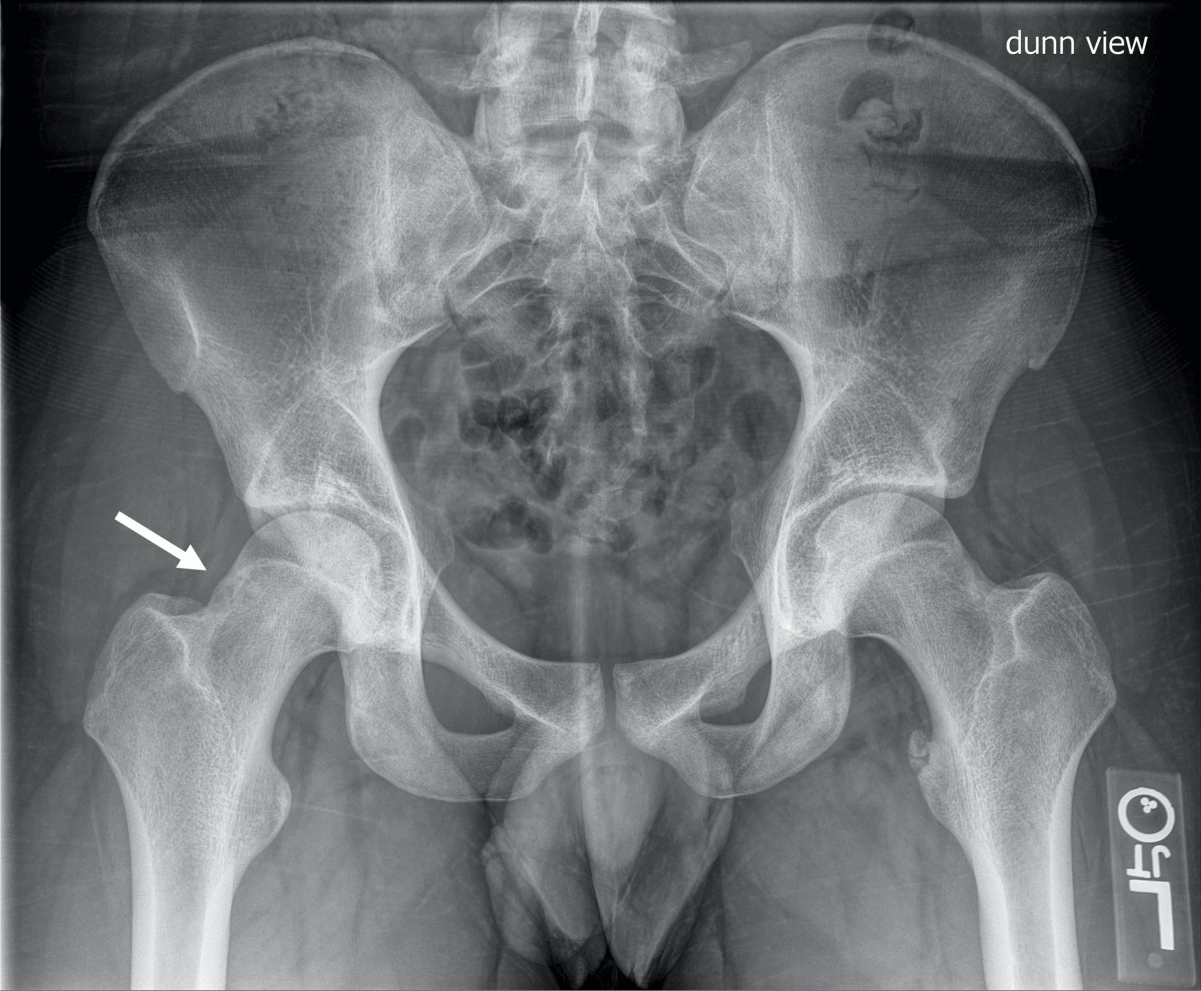
Pre-operative 45° Dunn lateral x-ray shows cam anatomy (arrow) of the right proximal femur with an alpha angle of 72°

Initial x-rays of the pelvis revealed a cam deformity on Dunn lateral radiograph with an alpha angle of 72° (Figure 1).

**Figure 2:**
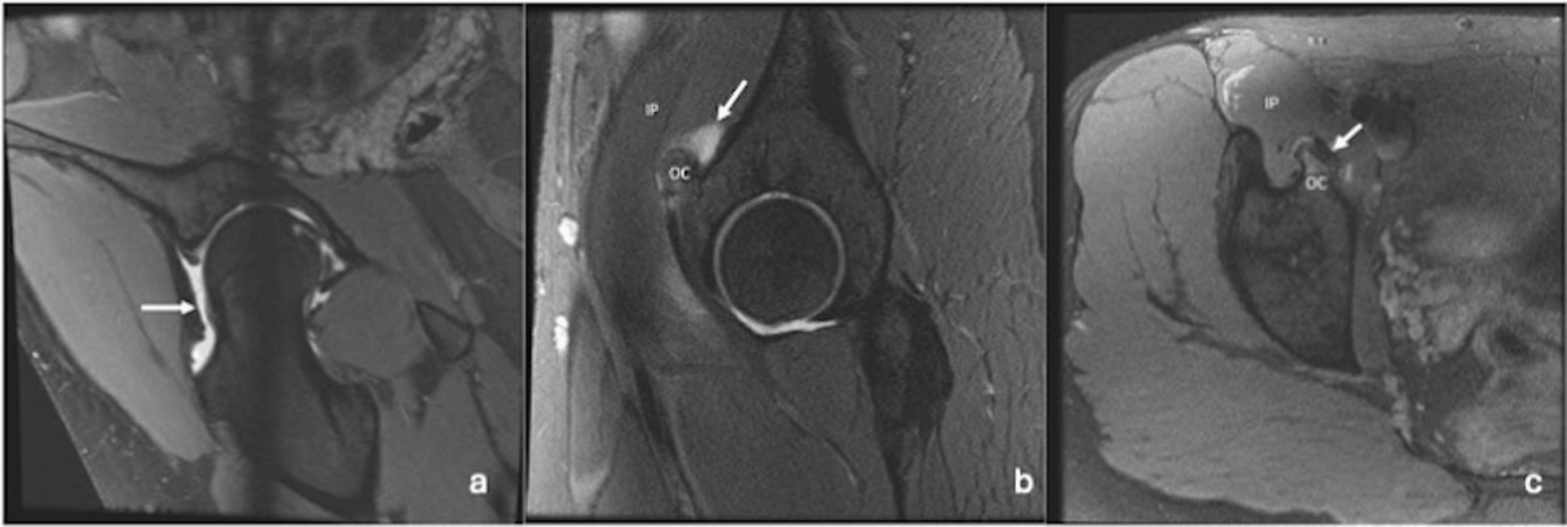
(a-c) Pre-operative MRA MRA: MR arthrogram; OC: osteochondroma; IP: iliopsoas muscle

Subsequent MR arthrogram (MRA) confirmed FAI anatomy, with a large cam deformity associated with a labral tear. Surprisingly, a pedunculated osteochondroma was found arising from the superior pubic ramus, abutting the psoas tendon, with associated edema within the adjacent psoas muscle and bursa (Figure 2).

Because it was unclear on physical exam how much of his pain was coming from the hip joint versus the iliopsoas, a diagnostic intra-articular injection of ropivacaine accompanied the MRA study. The patient reported 80% decrease in anterior hip pain following the injection, suggesting that intra-articular hip pain from cam FAI was responsible for a significant portion of his symptoms. However, the intra-articular injection did not alleviate his complaint of painful snapping hip symptoms. Given his exam and imaging findings, we concluded that his symptoms could be attributed to both intra-articular impingement from FAI and extra-articular pathology of chronic iliopsoas tendinitis from a snapping iliopsoas tendon over the superior pubic ramus osteochondroma. After discussion with the patient, who wanted both diagnoses to be addressed, we proceeded with right hip arthroscopy for labral repair and femoral osteochondroplasty, and open osteochondroma excision.

**Figure 3:**
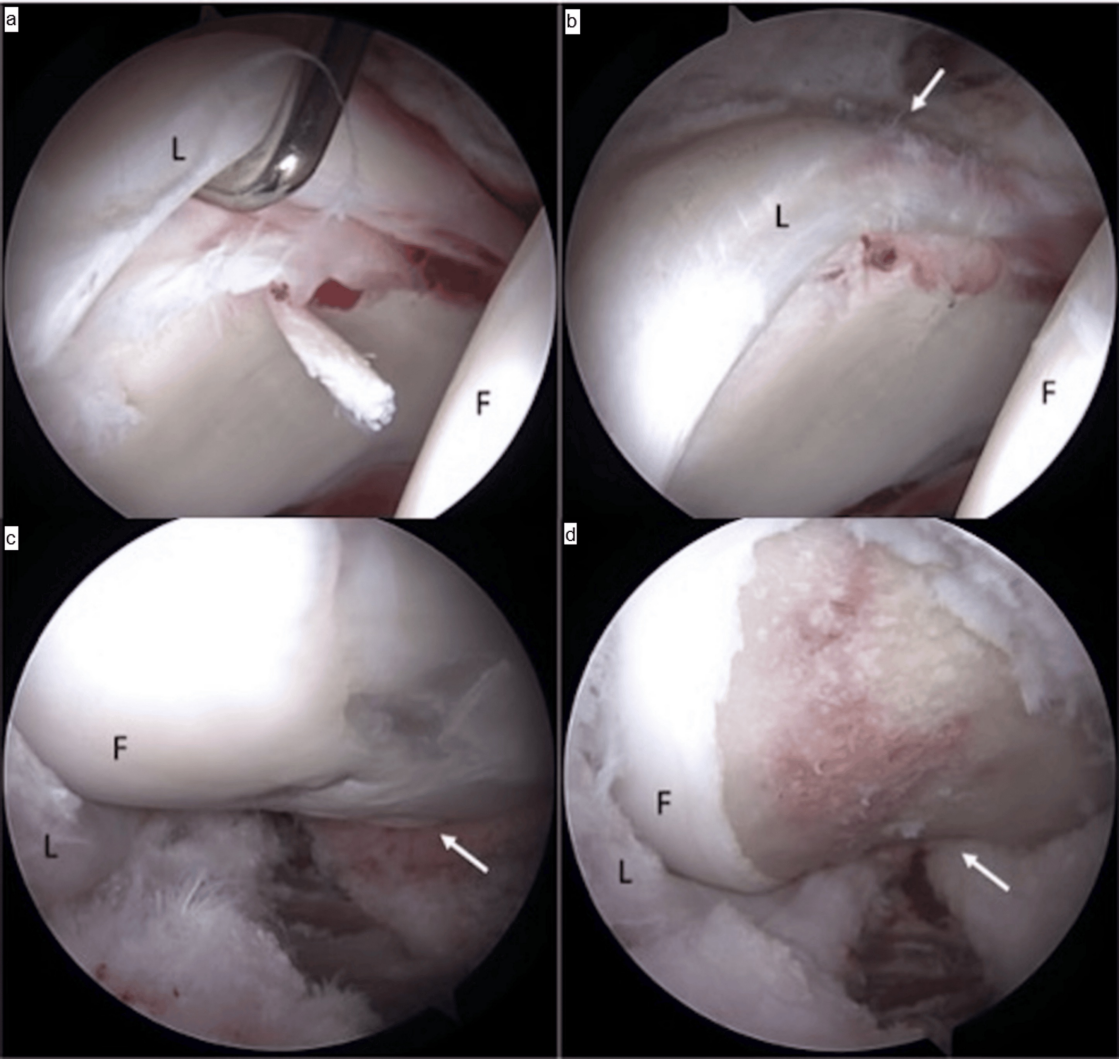
Arthroscopy images Intra-operative arthroscopic images demonstrating intra-articular pathology secondary to cam FAI, with acetabular labrum (L) and femoral head (F) in view. (a) View of the central compartment of the hip joint shows an unstable acetabular labrum with labrochondral separation as indicated by the arthroscopic probe. (b) After labral repair with suture anchors, normal labral stability was restored. (c) View of the peripheral compartment of the hip joint demonstrates a large cam deformity of the proximal femur (arrow). (d) After femoral osteochondroplasty to resect cam, femoral head-neck offset (arrow) was improved, and no further impingement was seen with flexion of the hip. FAI: femoroacetabular impingement

Hip arthroscopy was performed using Smith and Nephew leg positioners and post, utilizing standard anterolateral and modified mid-anterior portals with an inter-portal capsulotomy. A labrochondral separation was found from the 12 to 2 o’clock position (Figure 3).

The labrum was repaired using two Stryker Pivot NanoTack suture anchors in a mattress-type pattern. Following fixation, the labrum was stable with restoration of the labral seal. Femoral osteochondroplasty was performed with a 5.5 mm round burr under fluoroscopic guidance. We then closed the hip capsule.

**Figure 4:**
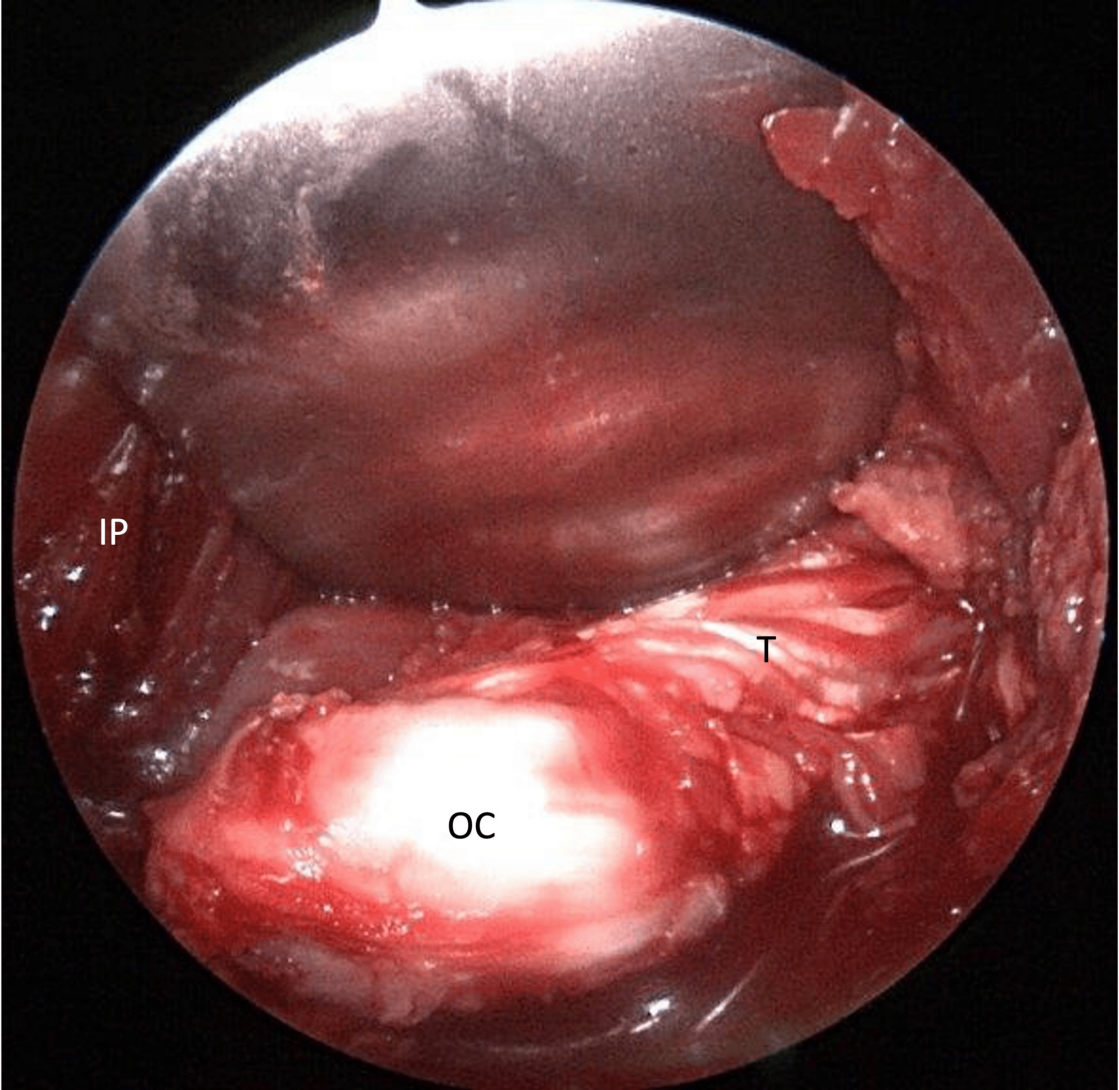
Osteochondroma (OC) excision An open approach to the superior pubic ramus exposed the OC, which was identified just lateral to the iliopsoas muscle (IP) and tendon (T). A portion of the T was seen draped over the stalk of the OC.

He remained supine with the right hip flexed in the leg positioner. We made a separate 5 cm bikini incision over the anterior aspect of the right hip and exposed the superior pubic ramus through the modified Hueter approach. The osteochondroma was palpable and embedded within the iliopsoas muscle. We carefully retracted the muscle over the cartilage cap to identify the stalk originating from the anterior limb of the triradiate cartilage near the superior pubic root. The cartilage cap of the osteochondroma measured approximately 1.5 cm by 1 cm with a height of 2 cm and was tensioning the iliopsoas tendon, which was directly medial to the osteochondroma (Figure 4).

**Figure 5:**
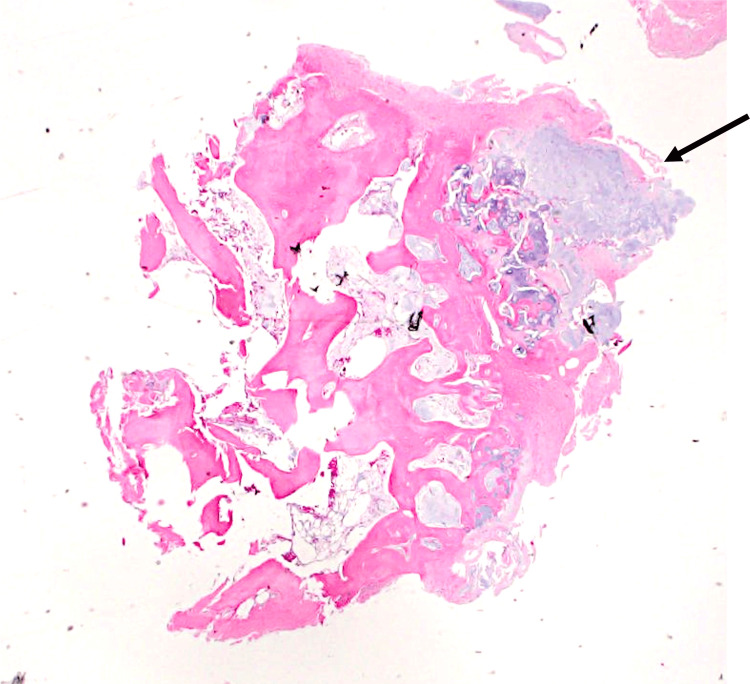
Histopathology of the lesion Photomicrographs of the osteochondroma specimen confirmed the diagnosis, demonstrating a cap of mature hyaline cartilage (arrow) atop a stalk of mature bone.

As we took the hip through a range of motion, the iliopsoas tendon rolled laterally over the osteochondroma. The osteochondroma was excised at the base of its stalk using an osteotome and rongeur, and excess bursal tissue was also removed. The osteochondroma was sent to pathology, which later confirmed the diagnosis (Figure 5).

**Figure 6:**
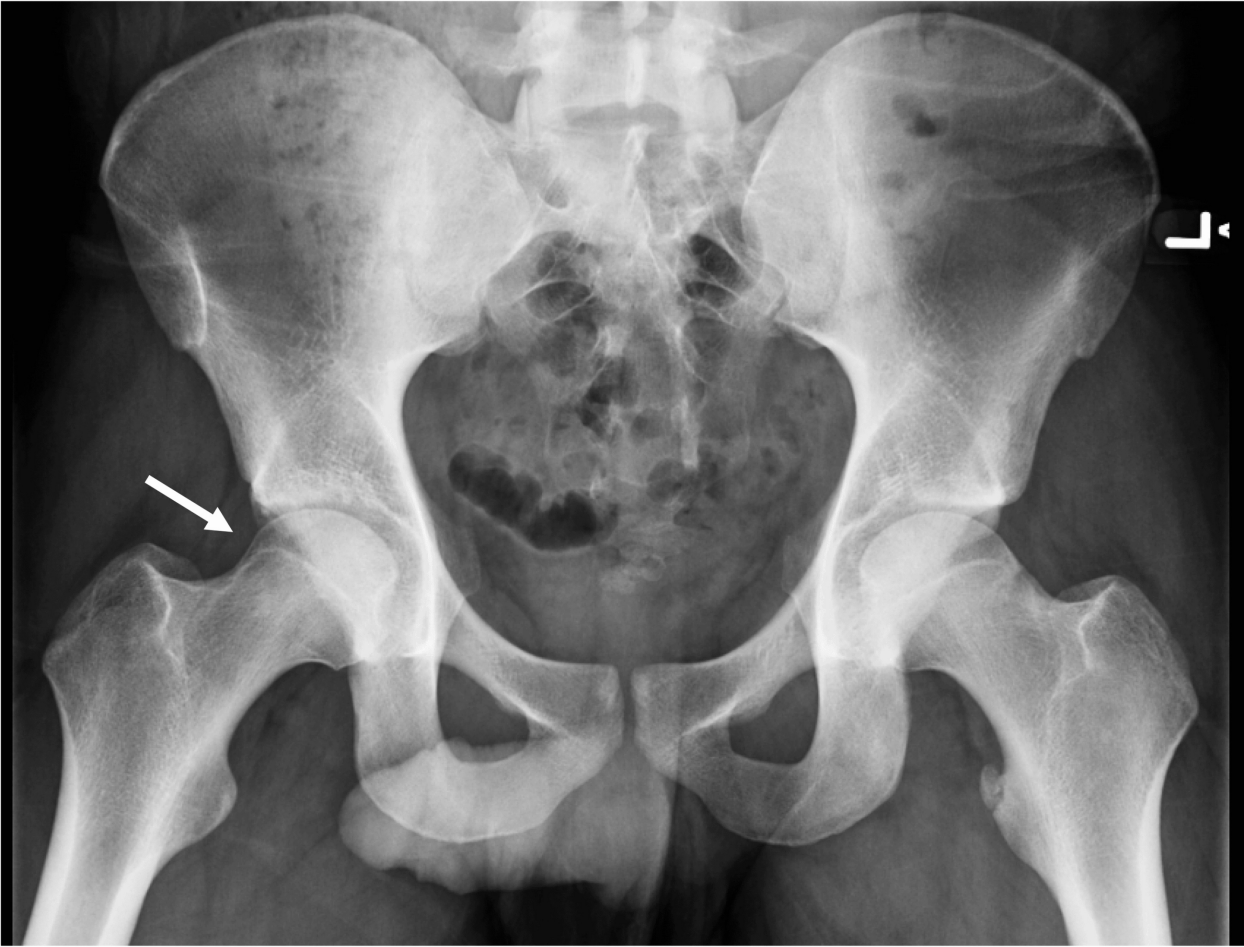
Post-operative x-ray Post-operative Dunn lateral x-ray at 7 months after surgery demonstrates correction of his cam deformity (arrow) with a decreased alpha angle of 46°. Joint space is well-preserved.

Post-operatively, the patient was one-sixth weight-bearing on the right lower extremity for two weeks, followed by progression to weight-bearing as tolerated. At his six-week post-operative visit, he had no hip pain and complete resolution of snapping. At the seven-month follow-up, he had returned to running and recreational football. He reported no hip pain except for occasional discomfort with prolonged sitting. His hip range of motion was from full extension to 95° of flexion, with 30° of internal rotation and 60° of external rotation in flexion. The Trendelenburg test and anterior impingement test were both negative. Radiographs demonstrated improved femoral head-neck offset, with an alpha angle of 46° (Figure 6).

Patient-reported outcome (PRO) scores at the one-year post-operative visit included a UCLA Activity Score [4] of 9 and Hip Disability and Osteoarthritis Outcome Score (HOOS) [5] subscores of 85 for Symptoms, 97.5 for Pain, 98.5 for Activities of Daily Living (ADL), 100 for Sport/Rec, and 87.5 for quality of life (QoL). He reported that he was “extremely satisfied” with his surgery and rated his hip at 82% of normal.

## Discussion

We describe a case of right hip pain due to a combined presentation of an unusual cause of internal snapping hip from an osteochondroma of the superior pubic ramus and symptomatic FAI secondary to a cam deformity. The patient underwent hip arthroscopy, labral repair, femoral osteochondroplasty, and open osteochondroma excision, which resulted in complete resolution of his symptoms.

Osteochondromas are the most common benign tumor of bone and are characterized by an osseous projection with an overlying hyaline cartilage cap [6]. Pelvic osteochondromas are uncommon, comprising approximately 5% of all osteochondromas [7]. Most case reports have described arthroscopic techniques for resection of femoral neck or acetabular osteochondromas [8-12]. Pelvic osteochondromas of the pubic ramus are extremely rare, with one case report of an 18-year-old woman who presented with groin swelling due to a large osteochondroma of the superior pubic ramus, which was treated with open excision. However, her complaint was primarily cosmetic, and she did not report symptoms of snapping hip nor FAI [13]. Chun et al. reported an osteochondroma of the anterior inferior iliac spine (AIIS) causing painful internal snapping of the iliopsoas tendon that was treated with open excision of the osteochondroma [14]. Although the location of this osteochondroma at the AIIS is close to the superior pubic ramus, this patient did not have concomitant symptoms of intra-articular hip pain from FAI.

To our knowledge, there are no described cases of an osteochondroma arising from the superior pubic ramus causing painful snapping of the iliopsoas tendon, with concomitant symptomatic FAI. A systematic review by Makhdom et al. identified 21 articles, all case reports, of osteochondromas of the femoral neck, acetabulum, or both. There was no consensus in the literature regarding the optimal surgical treatment, and the surgical approach was guided by the location of the lesions [15]. In our case, the location of the superior pubic ramus osteochondroma was less amenable to arthroscopic access, so a combined arthroscopic and open approach was utilized to address both causes of the patient’s hip pain: the FAI causing intra-articular hip pain and the osteochondroma causing the snapping iliopsoas tendon.

## Conclusions

This unusual presentation of intra-articular pain combined with extra-articular snapping hip demonstrates the diagnostic dilemma associated with anterior hip pain. Snapping hip should not be assumed as a symptom of FAI, and a careful workup is needed to determine the appropriate diagnoses and treatment. First-line treatment for both snapping iliopsoas tendon and FAI is a trial of physical therapy. If snapping hip does not improve with physical therapy, early advanced imaging such as CT or MRI can potentially reveal causative pathologies. Diagnostic injections can help identify the location of pain and differentiate which diagnosis is responsible for the majority of symptoms. In this case, some of the patient’s hip pain was caused by FAI, but he also had pain from a snapping iliopsoas tendon. Thus, the patient would have likely continued to experience painful snapping hip if only his FAI had been treated and the osteochondroma remained.

Although a painful snapping iliopsoas tendon occasionally presents as a symptom of FAI, clinicians should not presume it is a symptom of FAI, as other rare conditions such as pelvic osteochondroma can also cause snapping hip. Careful evaluation of anterior hip pain including advanced imaging and diagnostic injections may be needed for comprehensive treatment.
